# Implications of Changing Temperatures on the Growth, Fecundity and Survival of Intermediate Host Snails of Schistosomiasis: A Systematic Review

**DOI:** 10.3390/ijerph14010080

**Published:** 2017-01-13

**Authors:** Chester Kalinda, Moses Chimbari, Samson Mukaratirwa

**Affiliations:** 1 School of Nursing and Public Health, College of Health Sciences, Howard College Campus, University of KwaZulu-Natal, Durban 4001, South Africa; Chimbari@ukzn.ac.za; 2 School of Life Sciences, College of Agriculture, Engineering and Science, Westville Campus, University of KwaZulu-Natal, Durban 4001, South Africa; Mukaratirwa@ukzn.ac.za

**Keywords:** growth, fecundity, survival, intermediate host snails, schistosomes, schistosomiasis, temperature

## Abstract

Climate change has been predicted to increase the global mean temperature and to alter the ecological interactions among organisms. These changes may play critical roles in influencing the life history traits of the intermediate hosts (IHs). This review focused on studies and disease models that evaluate the potential effect of temperature rise on the ecology of IH snails and the development of parasites within them. The main focus was on IH snails of schistosome parasites that cause schistosomiasis in humans. A literature search was conducted on Google Scholar, EBSCOhost and PubMed databases using predefined medical subject heading terms, Boolean operators and truncation symbols in combinations with direct key words. The final synthesis included nineteen published articles. The studies reviewed indicated that temperature rise may alter the distribution, optimal conditions for breeding, growth and survival of IH snails which may eventually increase the spread and/or transmission of schistosomiasis. The literature also confirmed that the life history traits of IH snails and their interaction with the schistosome parasites are affected by temperature and hence a change in climate may have profound outcomes on the population size of snails, parasite density and disease epidemiology. We concluded that understanding the impact of temperature on the growth, fecundity and survival of IH snails may broaden the knowledge on the possible effects of climate change and hence inform schistosomiasis control programmes.

## 1. Introduction

Schistosomiasis is associated with high morbidity and mortality in sub-Saharan Africa (SSA) [1]. There are two main forms of the disease in SSA, urogenital and intestinal schistosomiasis [2], mainly caused by trematode parasites *Schistosoma haematobium* and *Schistosoma mansoni*, respectively [3,4]. Transmission of these parasites is dependent on many factors. They range from climatic factors such as rainfall and temperature [5,6] to IH (intermediate host) snails, parasites and human related factors that may maintain or increase the transmission of the disease [7]. Although praziquantel has been used to reduce infection among infected individuals, cases of re-infection due to exposure to infected water as well as tolerance of the parasite to praziquantel, have been reported in endemic areas [8,9,10]. Furthermore, a rise in temperature due to climate change may modify the incidences of schistosomiasis by altering the snail fecundity, growth and survival rates, their distribution patterns and parasite development rate [11,12]. It is therefore important to understand the temperature-driven changes on the snail and snail-parasite system to enhance our knowledge on the climate-disease theory.

Growing evidence suggests that climate change will lead to an increase in temperature [13,14] and incidences of extreme events [15]. The predicted changes in temperature are expected to impact on the ecological interactions between organisms thereby affecting possible outcomes of host-parasite interactions [16,17]. Studies on the implications of climate change on diseases have mainly been done for malaria [18,19,20] with only a few focusing on schistosomiasis [2,21,22]. Thus, there has been a continuous debate on the extent to which schistosomiasis burden will be influenced by climate change. Therefore, this necessitates further investigations to identify the major stages within the IH life cycle that are likely to be impacted most.

Significant progress has been made towards understanding the epidemiology and control of schistosomiasis [23,24]. This has led to initiatives such as mass drug administration [25,26], the development of schistosomiasis control programmes [27,28] and disease epidemiology and GIS (Geographic Information System) models [29,30,31,32]. However, the projected impact of climate driven changes on the epidemiology of schistosomiasis may compromise the control efforts. Although some snail interruptive measures such as the use of molluscicides and biological control have been used [33,34], the influence of temperature on snail population size and parasite distribution is not well understood for the efficient management, surveillance, control and prevention of the disease [32]. Understanding the role of temperature in driving changes in the ecology and population dynamics of both infected and non-infected snails is essential for predicting the possible impacts of climate change on future schistosomiasis dynamics [35]. This information can be obtained from evidence based field and laboratory studies. The present review therefore explored the effects of temperature on the growth, fecundity and survival of IH snail species from the genus *Bulinus* and *Biomphalaria*.

## 2. Materials and Methods

### 2.1. Search Strategy

A systematic search of literature on Google Scholar, PubMed and EBSCOhost databases was conducted using the following terms and Boolean operators (OR, AND): Schistosomiasis AND Temperature, *Bulinus* OR *Biomphalaria* AND growth, fecundity AND survival in sub-Saharan Africa. Articles that were identified were screened by reading through the titles and abstracts. In addition, reference and bibliographic lists of the selected articles were screened as potential leads to any additional relevant studies for inclusion. Full text articles were retrieved and managed in Endnote reference manager version X7 (Clarivate Analytics, Philadelphia, PA, USA). The review included literature from 1980 to 2016.

### 2.2. Inclusion Criteria and Exclusion Criteria

**Figure 1:**
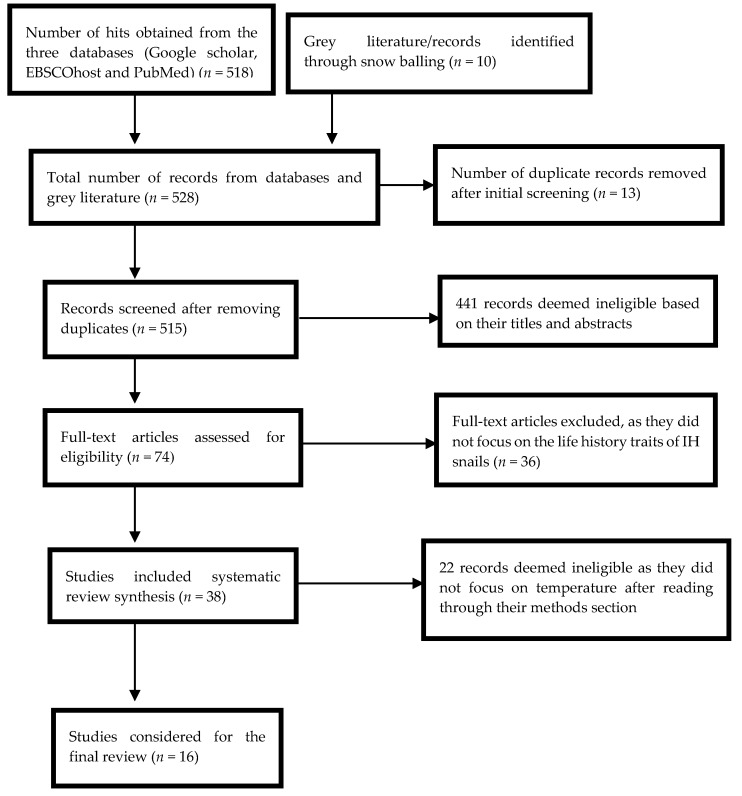
PRISMA diagram.

Studies were included in the review if they were published in peer-reviewed journals and explicitly reported on (1) temperature and IH snails of schistosomes that cause human schistosomiasis and (2) the genus *Bulinus* or *Biomphalaria* (Figure 1, Table 1).

## 3. Results

The systematic literature search yielded a total of five hundred and twenty-eight hits which included abstracts, reports, books and duplicate articles (Figure 1). Four hundred and ninety articles were excluded due to duplication and did not explicitly report on temperature in relation to *Bulinus* (hereafter abbreviated as *Bu.*) and *Biomphalaria* (hereafter abbreviated as *Bi.*) snail species as the IH of schistosomes that cause human schistosomiasis. A further twenty-two articles were excluded as they did not focus on temperature related studies although they looked at the life history traits of IH snails. Sixteen publications were considered for this review (Table 1). The results from the publications that were eligible for the review were classified into the following themes: temperature and timing of sexual maturity and fecundity, temperature and growth, temperature and survival and temperature and parasite development. These are discussed below in relation to potential change in the disease risk with a possible rise in temperature.

### 3.1. Temperature and Timing of Sexual Maturity and Fecundity

Temperature has been observed to have variable effects of the timing of sexual maturity and fecundity of both *Bulinus* and *Biomphalaria* snail species. According to Barbosa et al. [46], the reproductive rate of *Bi. glabrata* varied inversely with temperature. At 24 °C, the reproductive rate was reduced while it was maximal at 19.9 °C. El-Emam and Madsen [38] working independently on *Bi. alexandrina* reported that the net reproductive rate was optimal at 26 °C. A study by Appleton and Eriksson [36] suggested that above 27 °C, *Bi. pfeifferi* had reduced egg mass production. In another study, McCreesh et al. [41] concluded that at 21.6 °C, the fecundity of *Bi. sudanica* was at its optimal. *Bulinus* species have been observed to tolerate higher fecundity temperature levels. According to Kubiriza et al. [40], the number of eggs laid by *Bu. nyassanus* maintained at 22, 25, 28 and 31 °C were not significantly different. However, at 22 °C, the net reproductive rate of the snails was greatly reduced (Table 2).

### 3.2. Temperature and Growth

Growth has been observed to be influenced by temperature. According to Kubiriza et al. [40], the shell heights of *Bu. nyassanus* maintained at 25, 28 and 31 °C were not significantly different. However, snail growth was reduced at 22 °C. Woolhouse and Chandiwana [48] working on *Bu. globosus* in Zimbabwe observed that the shell height of snails increased linearly with temperature. Snails that were maintained at 22.5–23.5 °C had longer shell heights than those maintained at 14–16 °C. Furthermore, O’keeffe [47] suggested that their intrinsic growth rate reduced if these snails were maintained at temperatures above 28.5 °C. Although increased snail growth rate had been observed to increase with temperature, a study by McCreesh et al. [41] suggested that the relationship between water temperature and the growth rate of *Bi. sudanica* was not clear.

### 3.3. Temperature and Survival

The survival of snails at various temperatures may be key to the establishment and invasion of snails into new areas. *Bulinus* species have been observed to tolerate higher survival temperatures than *Biomphalaria* species. According to Dagal et al. [37], the maximal survival of *Bu. abyssinucus* was observed to be in the temperature ranges between 20 and 35 °C while no snails survived at 40 °C. Kubiriza et al. [40] suggested that the survival of *Bu. nyassanus* was optimal at 25 °C. However, this was not significantly different from the survival of snails at 28 and 31 °C. Joubert et al. [39] observed that *Bu. globosus* had marked increases in survival at higher temperatures (34 and 36 °C) as compared to *Bi. pfeifferi* and *Bu. africanus.* El-Emam and Madsen [38] observed that the survival of *Bi. alexandrina* and *Bu. truncatus* was reduced at temperatures above 33 °C and below 10 °C. According to McCreesh and Booth [50], the survival of *Bi. pfeifferi* outside the temperature ranges of 14.0–31.5 °C is greatly reduced, leading to a possible reduction in disease risks. Infected *Bi. glabrata* maintained at 16 °C were observed to have a reduced survival rate [43], while the survival of infected *Bu. truncatus* was also observed to be reduced at 17 and 33 °C [42].

### 3.4. Temperature and Parasite Development

Temperature has been observed to influence the parasite development rate. According to McCreesh and Booth [50], the prepatent period of *Bi. pfeifferi* reduced from 130 days when maintained at 14 °C to 18 days when maintained at 32 °C. On the other hand, Pflüger [43] observed that *Bi. glabrata* snails maintained at 32 and 33 °C had a prepatent period of 15 days. Furthermore, the study suggested that below 14.2 °C, the development of cercariae in *Bi. glabrata* comes to a standstill. Pflüger et al. [42] also observed that *Bu. truncatus* snails maintained at 18 °C had the longest prepatent period of 106–113 days while it was 17–19 days for snails maintained at 30–31 °C.

### 3.5. Temperature Dependent Model Formulation

A number of schistosomiasis models, mathematical and statistical have been developed to understand the transmission dynamics of schistosomiasis. Most of these models synthesize information from experimental studies and extrapolate to give future projections.

#### 3.5.1. Statistical Models

Prediction of the distribution of snails has been used to potentially indicate the risks of disease transmission. Studies by Pedersen et al. [52] and Stensgaard et al. [53] have combined aspects of temperature and Geographic Information System (GIS) to predict the spatial distribution of the disease. These studies reveal that areas that will be suitable for both snail and parasite distribution. The studies further suggest that variable habitats may be created due to rise in temperature.

#### 3.5.2. Mathematical Models

A model by McCreesh and Booth [50] suggested that in the two main schistosome transmission sites, rivers and lakes, transmission may be high at 15–19 °C and 20–25 °C, respectively. The model further suggested that a rise in temperature may reduce the disease risks in some transmission zones such as lakes, ponds, reservoirs and dams. Furthermore, that temperatures around 20 °C may lead to an increase in disease risks in rivers and streams while the risk of infection is likely to reduce at temperatures above 25 °C.

Ngarakana-Gwasira et al. [51] suggested that the temperature range between 18 and 28 °C was the most ideal for disease transmission. The model further suggested that 23 °C was the most ideal temperature for disease transmission. The model by Mangal et al. [49] suggested that schistosomiasis prevalence would be stable in the temperature range of 20–35 °C. The model also suggested that the increase in the survival of the parasite at 20 °C indicated that this was the most ideal temperature for applying parasite control measures.

## 4. Discussion

### 4.1. Growth and Fecundity Components: Do Temperature Levels Matter?

Snails allocate their resources towards growth, fecundity and survival [54] and the allocation of resource to any of these traits comes as a trade-off between growth, fecundity and survival [55,56]. The allocation of resources to fecundity means a reduction in resource allocation to the other physiological functions, growth and survival. Temperature on the other hand affects the physiological function of organisms [17,57]. It plays an important role in the timing of sexual maturity in IH snails [21,36]. High temperatures promote increased egg mass output and hastenens gametogenesis [58]. Therefore, this suggests that a rise in temperature may have a positive effect on snail fecundity as well as snail population growth. On the other hand, temperature levels above 30 °C have been observed to affect gametogenesis [22], suggesting that the positive effect of temperature on snail fecundity can only occur within a certain temperature range thermally tolerable to snails. Determination of this temperature range is important for understanding the likely impact of climate change on the population dynamics of snails in order to inform snail control programmes [49].

Studies done by McCreesh et al. [41], Kubiriza et al. [40] and El-Emam and Madsen [38] suggested that temperature strongly enhanced the fecundity of IH snails. In another study, Barbosa et al. [46] suggested that the relationship between reproductive rate of *Bi. glabrata* and temperature was non-linear. These studies have shown that different snails species have different optimal temperatures for fecundity. The output of egg masses at various temperature levels may also suggest that some IH snail species may be well adapted to low or high temperatures. Production of more egg masses at high temperature may also suggest that a possible rise in temperature may lead to an increase in the population size of IH snails especially during the post-rainy and cold dry seasons [59]. This may have implications on the incidences and prevalence of schistosomiasis. McCreesh et al. [35] have also suggested that a rise in temperature may potentially increase habitats suitable for transmission of *S. mansoni*. The study further observed that this may lead to increased risks of disease transmission in Rwanda, Burundi, south-west Kenya and the eastern parts of Zambia. This may be attributed to an increase in the population size of IH snails. Earlier studies by Appleton [60] and Michelson [22] while working on *Bi. pfeifferi* and *Bi. glabrata,* respectively, observed that the optimal fecundity temperature range for these snails was higher than that observed by McCreesh et al. [41]. This suggests that *Biomphalaria* species may have a slightly higher optimal temperature for fecundity and hence rise in temperature may alter the population dynamics and schistosomiasis incidences.

A recent snail survey in Uganda observed that snails may be found at high altitudes [61]. This may suggest that temperature may have increased, thus creating new habitats for snail invasion in places previously observed to be unsuitable for snails. This may also translate into the spread of the disease to areas that previously were unsuitable for disease occurrence.

High temperatures have also been associated with altered membrane permeability, increased accumulation of toxic metabolites and reduced immunity defences of IH snails [37,62]. This may increase the vulnerability of snails to infection which may later affect the survival and egg mass output [54,63,64]. Studies exploring the fecundity responses of *Bulinus* species when exposed to different temperature levels concluded that these IH snails maintained maximal reproductive output at 25 °C [65,66]. *Bulinus globosus* snails have also been observed to be highly fecund, tolerant to high temperatures and to re-populate quickly [39,47,65]. Thus, the results from these studies suggest that egg mass output at supra-optimal temperatures and the optimal fecundity temperature for *Bulinus* species may be lower than that observed by Kubiriza et al. [40].

The results from the reviewed studies may be insightful in predicting the possible impacts of temperature rise on snail fecundity. However, besides differences in the snail species used in the laboratory experiments, methodological differences ranging from the source of IH snails used (field or laboratory bred snails) and their acclimatization period to laboratory conditions (field collected snails) may have affected the performance of snails in the various studies. For example, the studies by Kubiriza et al. [40] and McCreesh et al. [41] both used field collected snails while Pflüger et al. [42] used a cohort of both field collected and laboratory bred snails. The likely exposure of field collected IH snails to more than two temperature regimes (field temperature, acclimatization temperature and experimental temperature) may have had an impact on the physiology of the IH snails and ultimately their performance. Mofolusho and Benson [44] conducted a study to evaluate the effect of acclimatization of field collected *Bi. pfeifferi* (Krauss, 1848) to laboratory conditions on snail survival and fecundity. The study observed that the survival and fecundity of acclimatized field collected IH snails were much lower than laboratory bred snails. Furthermore, Paull et al. [14] suggested that the use of field collected IH snails in laboratory temperature driven experiments after their prior exposure to field temperatures (high or low) may affect their future physiological functions. This is because temperature is energetically stressful on IH snails and may affect their physico-chemical conditions [14,67]. This may therefore lead to a reduction in their performance due to depleted energy resources in a new environment or an increase in their performance as a result of increased energy allocation to one trait due to change in temperature. This also suggests that the use of results from laboratory studies based on field collected snails should be done with caution, taking into account the limitations.

Besides fecundity, snail growth is another important life history trait that can determine the invasion and adaptation of snails to new areas. Growth affects the fecundity, ability of IH snails to withstand infection and availability of resource to trematodes during intramolluscan trematode development [21,48,68]. According to McCreesh et al. [41], the relationship between water temperature and IH snail growth was unclear. Other studies [38,40] observed an increase in the growth of IH snails with a rise in temperature (Table 1). Although the initial sizes of IH snails used by McCreesh et al. [41] varied and snails of different sizes were also used in the study by Kubiriza et al. [40], it is evident that temperature enhances snail growth. Studies conducted by Appleton [60] and Michelson [22] suggested that higher temperatures accelerate the growth of IH snails compared to lower temperatures. It was observed that *Bi. pfeifferi* had optimal growth at temperatures between 22.8 and 28 °C while *Bi. glabrata* snails maintained at 30 °C had accelerated growth compared to those at 25 °C. Furthermore, the optimal temperature ranges observed from these studies coincide with the optimal disease transmission temperature range (22–27 °C) [66]. This may suggest that increased snail population and growth within this temperature range may lead to a possible rise in the prevalence of schistosomiasis although this can only happen within temperature ranges tolerable by snails. Growth is a physiological trait; hence it can only increase within a thermally favourable temperature. Above this temperature range, no further growth may be observed. At extreme temperatures, the growth of snails diminishes and mortality rates are high [51,64].

### 4.2. Snail Survival and Temperature Rise: Its Role in Schistosomiasis Incidence

Host survival is an important aspect for the success of completing the disease transmission cycle. The survival time of IH snails is affected by both biotic and abiotic factors [5]. Infection of IHs has been observed to lead to phenotypic modifications which affect their survival time [69]. The development of schistosomes and the completion of their life cycle within the IH snails is greatly dependent on the survival of snails [70]. Survival of various IH snails has been observed to occur at various temperatures. Non-infected *Bi. sudanica* had optimal survival at 20 °C [41]. Temperatures below or above this increased snail mortality. For non-infected *Bu. globosus*, low and high temperature studies [39,45,47] concluded that these snails were well adapted to higher and lower temperatures compared to *Bi. pfeifferi*. O’keeffe [47] observed that *Bu. globosus* could survive at 30 °C and this was corroborated by the findings of Joubert et al. [39]. A study conducted by Appleton [60] concluded that the survival time of *Bi. pfeifferi* was significantly reduced when maintained above 27 °C. According to Joubert et al. [39], *Bu. globosus* had a lower mortality rate at 34 °C compared to *Bi. pfeifferi* suggesting that in some places, urogenital schistosomiasis may be more prevalent than intestinal schistosomiasis.

The role of trematode infection on snail survival time has had variable conclusions. Studies have shown that at high temperatures, mortality is high for both infected and non-infected IH snails [37,42,64]. Other studies [63,64] observed that infection had no effect on the survival time of IH snails, while Stirewalt [11] proposed that the mortality rates of infected IH snails rose with increasing temperature. This suggests a possible reduction in the amount of cercariae produced and in disease risks during dry hot seasons when the population size of snails is reduced [59].

Studies [38,39,40,41,45] have given insightful results on the effect of temperature on the survival of IH snails and its implications for disease transmission. However, most of the studies were done on non-infected snails. Understanding the thermal behaviour of both infected and non-infected snails may be important in predicting the overall effect of climate change on schistosomiasis. For example, within certain temperature levels, Studer et al. [64] observed that infected IH snails had higher survival rates compared to non-infected snails. A study by Seppälä and Jokela [62] suggested that temperatures above 30 °C affect the immunity defences of IH snails and increases their vulnerability to infection and infection induced mortality [71]. The observed variations may suggest the need for further studies on the interaction of temperature and infection on IH snail mortality. This may also assist in providing evidence to show if the alterations in the development of snails at high temperature is a result of phenotypic adaptation [62] and to estimate the possible effects of climate change on disease risks. This may also help develop more generalizable effects of temperature change on the population dynamics of IH snails and contribute to refined parameter estimations that may be included in disease models.

### 4.3. Schistosoma Development in Infected Snails: Does Temperature Matter?

The life cycle of *Schistosoma* parasites is complex as it involves molluscs and warm blooded mammals. The parasite also undergoes both asexual and sexual reproduction [2]. Once the parasite has been released into an aquatic environment by a mammal, the parasite must find a compatible IH snail species or it dies [2]. Temperature is key in that it affects the infectivity and the size of snails [63] influencing the amount of resources that may be available during the intramolluscan stages [54,68]. The intramolluscan parasite development stages are dependent on temperature [11,72,73] although Morley and Lewis [74] suggest that temperature may have no effect on the rate of cercariae development in infected hosts. Studies by Paull and Johnson [63] and Stirewalt [11] suggested that temperature influenced the rate of intramolluscan cercariae development. High temperatures have been observed to shorten the prepatent period [11,72]. Although low temperature may inhibit the development of cercariae [11,43,63], a rise in temperature to levels above the minimum cercariae development threshold temperature may lead to the production of cercariae [63]. This may also suggest that disease risks may be reduced during cold seasons despite the high IH snail population size. On the other hand, resumption of suitable conditions for cercariae development may lead to an increase in disease risks in such areas.

High temperatures elevate the output of cercariae [75] suggesting a possible increase in disease risks with a rise in temperature. On the other hand, very high temperature (i.e., 33 °C) and low temperatures (i.e., 17 °C) may lead to a reduction in disease risk due to increased snail mortality and a reduction in the development of cercariae within the IH snails [42,76]. This suggests that at extremely high temperatures such as those observed during the dry hot seasons, a reduction in disease incidence because of an increase in the mortality rate of both infected and non-infected IH snails may be experienced [64,70].

### 4.4. Disease Models Parameterization: Does Experimental Data Source Matter?

Following the initial model by Macdonald [77], a number of models have been developed to understand the transmission dynamics of schistosomiasis. Such models mainly focused on predicting disease transmission and intensity as well as providing solutions towards the application of chemotherapy [78,79,80].

#### 4.4.1. Statistical Models

Statistical models have been used to understand the risk factors of schistosomiasis and in developing relationships between various factors related to disease transmission [81]. This may contribute to improving disease monitoring and control through well designed disease and IH snail control programmes [78,79,80]. Parameterized statistical models have also been used to predict the possible estimates for disease threshold cases, snail population and worm burden that can be used in mathematical models to develop possible control measures [29,78]. Keeling and Rohani [82] proposed that variations in the ecology of IH snails that may be caused by temperature, may need to be accounted for in modelling. These variations may range from the growth, fecundity and survival rates to parasite production rates. For example, a schistosomiasis model by Liang et al. [83] fitted using least-squares modelled snail recruitment and showed how this is dependent on temperature. The model however assumed a constant snail mortality rate which may have led to the under-estimation or over-estimation of the effect of temperature on the snail population size. This assumption may also have disregarded the possible heterogeneity in the survival of IH snails maintained at different temperature with different infection statuses. Modelling has incorporated aspects of GIS to understand the effects of temperature on the transmission of schistosomiasis. Models by Pedersen et al. [52] and by Stensgaard et al. [32] concluded that temperature is an important factor that can limit the occurrence of schistosomiasis and creation of new snail habitats. Studies [39,47,84] observed that snail species such as *Bu. globosus* survived at 30 °C while *Bi. pfeifferi* exposed to temperatures above 29 °C experienced hyperthermia [60].

#### 4.4.2. Mathematical Models

The use of mathematical models has increased the understanding of the dynamics of schistosomiasis transmission and the implementation of IH control programmes [80]. It is however important that parameterized models take into account the important life history components of both the IH snails and the schistosome. A mechanistic model by Mangal et al. [49] describes the parasite burden, density of uninfected snails and those in the prepatent and patent stages. The model suggested that the mean worm burden was lowest at 20 °C, highest at 30 °C and diminished at 35 °C. The observed reduction in the worm burden at 35 °C suggests that high temperatures, especially those observed during dry hot summers may reduce prevalence of schistosomiasis [59]. A sensitivity analysis conducted on the model parameters showed the effectiveness of carrying out parasite control measures when temperature was about 20 °C. The model further suggested that application of chemotherapy at this temperature may reduce the parasite burden and prevalence of the disease. The model also shows how slight increases in temperature can affect the prevalence of schistosomiasis. However, the development and parameterization of the mechanistic model was based on non-species-specific *Biomphalaria* snail population.

Like the mechanistic model, a mathematical and GIS model for Zimbabwe has been developed by Ngarakana-Gwasira et al. [51]. The model explores the impact of temperature and other climatic factors such as rainfall on schistosomiasis transmission. The model suggests the ideal temperature range for schistosomiasis transmission and incorporates GIS to predict areas that may be vulnerable to disease spread with a rise in temperature. The conclusion from the mathematical and GIS model by Ngarakana-Gwasira et al. [51] has been based on non-specific snail species. Although the model was parameterized based on the works done on *Bulinus (Physopsis) abyssinucus* [37], this snail is little known as an IH of trematodes in Zimbabwe. The model however has observed that certain areas in Zimbabwe may need special attention owing to their potential for increased disease incidences. Areas such as Chiredzi and Mushandike have been observed to be high risk areas for disease transmission.

The agent based model by McCreesh and Booth [50] reported infection risks from river and lake scenarios based on three different temperature ranges. The study suggested the optimal temperature ranges for human infections at two possible schistosome infested points (lake or river). The model also showed the times of day at which cercariae shedding would be at its maximal level, thus demonstrating the importance of temperature in schistosome transmission. Furthermore, the study suggested that a rise in temperature may increase disease risks owing to increased cercariae output. This model agrees with the conclusion reached through a meta-analysis by Poulin [75]. The agent based model quantifies disease risk based on the amount of cercariae shed by snails into the natural environment. However, it does not take into consideration the effect of temperature on snail mortality and its eventual impact on the amount of cercariae that can be produced. This is because a rise in temperature increases snail mortality which may later impact on the amount of cercariae produced [64,74]. Furthermore, increased parasite induced mortality among infected snails [85] has been observed and this leads to a reduction in the population density of parasitized IH snails and possible amount of cercariae produced. However, some of the shortcomings of such models have been accounted for through the development of Bayesian models which include geostatistical and spatial scan statistics [81]. This makes statistical models useful in estimating parameters that can be used for the development of mathematical models.

### 4.5. Potential Future Research Areas

This review has shown that differences in the IH snail species used and in the methodological approaches may explain the variations observed from the various temperature driven studies. The impact of climate change on schistosomiasis still remains unclear owing to the many factors that may be at play. The distribution of schistosomiasis may not only be affected by temperature but also by the availability of water. Other factors such as IH snails, parasites and human related [7] may also affect disease distribution. This suggests that differences in the rainfall patterns and temperature in different areas may affect the fecundity, survival and growth of IH snails which ultimately impact on disease incidence. It is possible to evaluate the effects of temperature rise on disease incidences and snail ecology. However, confounding factors in different environments, exposure of IH snails to multiple variables, such as turbidity and pH among others, may also have an influence on the data obtained. Nevertheless, carefully designed laboratory experiments can provide data for parameterization of disease models.

There is a need to understand the effect of temperature on all the development stages of the snail-parasite system as well as the survival rates of IH snails over time. There may also be a need to evaluate the tolerance of successive snail generations to various temperature levels. This is because over time, snails may adapt to higher temperatures thus affecting the parameters used in disease models based on the performance of a single generation. To achieve this, the use of experimental data coupled with field studies may prove valuable in observing the changes in the environment. Temperature driven experiments based on infected and non-infected snails may assist in understanding the life stages that are likely to be affected the most by increasing temperature and how this will affect the dynamics of schistosomiasis. The use of GIS in models will also assist in projecting the likely areas that can be future disease hot spots owing to temperature increase.

## 5. Conclusions

The review shows that temperature is an important factor in the spread of schistosomiasis. It also shows that it may continue to play a critical role in designing schistosomiasis control programmes. Climate change may lead to a rise in temperature and this may lead to a complex relationship between IH snails and schistosomes. Although a rise in temperature may most likely lead to the disappearance of schistosomiasis in certain areas, a possible increase in the snail population due to a rise in fecundity and a reduction in the parasite development rate may increase schistosome infective stages in other areas. Disease modelling, mathematical and statistical, may however be a useful tool in understating the epidemiology of schistosomiasis. However, these models may need to include most temperature dependent stages and the biology of the IH snails and schistosomes for improved predictions, analysis and application. The use of data from well-designed laboratory and field experiments that systematically assess the effect of temperature at various stages of the snail and parasite development, will also improve the precision of models.

## Figures and Tables

**Table 1:** Summary of laboratory studies, field studies and models that assessed the effect of temperature on *Bulinus* and *Biomphalaria* snail species between 1980 and 2016.

Author (Reference)	Objective	Snail Species Studied	Methods	Outcome
Appleton and Eriksson [36]	To determine the influence of fluctuating above-optimal temperature regimes on the fecundity of *Bi. pfeifferi*	*Biomphalaria pfeifferi*	Laboratory experiment	Snails with the shell height of 1.5–2.5 mm did not produce egg masses and had the least survival rate. Fecundity was observed to reduce when snails were exposed to temperature above 27 °C.
Dagal et al. [37]	To determine the effect of some physico-chemical factors (temperature, pH and salinity) on the hatchability of egg masses and survival of juvenile and adult snails	*Bulinus (Physopsis) abyssinucus*	Laboratory experiment	No eggs hatched at 5, 10 or 15 °C. The mean survival rate of snails at 5 and 10 °C was zero. Best egg hatching temperature was 25 and 30 °C. No eggs hatched at 35 °C. Maximal snail survival time was observed at temperatures between 20 and 35 °C. No snail survived at 40 °C.
El-Emam and Madsen [38]	To compare the effect of temperature on the growth, survival and fecundity of *Bu. truncatus* and *Bi. alexandrina*	*Biomphalaria alexandrina* and *Bulinus truncatus*	Laboratory experiment	Minor differences in the shell height of *Bi. alexandrina* and *Bu. truncatus* maintained at 26 °C and 28 °C. The survival of *Bi. alexandrina* and *Bu. truncatus* greatly reduced at 10 °C and 33 °C. For both snail species, the net reproductive rate was optimum at 26 °C.
Joubert et al. [39]	To determine the survival of *Bu. africanus* (Krauss), *Bu. globosus* and *Bi. pfeifferi* at constant high temperatures of 34 °C to 40 °C	*Bulinus africanus* (Krauss), *Bulinus globosus* (Morelet) and *Biomphalaria pfeifferi* (Krauss)	Laboratory experiment	Marked increase in the survival of *Bu. globosus* observed at 36 °C and 34 °C. The survival time of *Bu. globosus* at 34 °C was almost four times longer than the survival of *Bu. africanus* and *Bi. pfeifferi*. Higher temperatures were more favourable for *Bu. globosus* than *Bu. africanus* and *Bi. pfeifferi*.
Kubiriza et al. [40]	To compare the performance (survival, growth, hatchability and reproduction) of *Bu. nyassanus* to other *Bulinus* spp. when exposed to different constant temperatures	*Bulinus nyassanus* (Smith, 1877)	Laboratory experiment	No significant differences in the growth of snails maintained at 25, 28 and 31 °C but slowest at 22 °C. No significant differences in the survival rate of snails at 25, 28 and 31 °C. However, it was observed to be optimal at 25 °C. No significant differences in the mean number of eggs laid across temperatures. The net reproductive rate was greatly reduced at 22 °C compared to the other three temperatures.
McCreesh et al. [41]	To determine the effects of water temperature on the mortality, fecundity, and growth rates of *Bi. sudanica*	*Biomphalaria sudanica*	Laboratory experiment	Water temperature strongly affected snail fecundity. Optimal snail fecundity (maximum mean number of eggs/snail/week) was observed at 21.6 °C. Very low and very high temperatures reduced snail survival. Least snail mortality was observed at 19 °C. No clear relationship between water temperature and snail growth. However, for the small and medium sized snails, fastest growth was observed at 23.1 °C and 23.3 °C, respectively.
Pflüger et al. [42]	To evaluate the effect of temperature on the development rate of *Schistosoma haematobium* development in *Bulinus* snails	*Bulinus truncatus*	Laboratory experiment	High snail mortality observed among snails maintained at 17 and 33 °C. Surviving snails did not develop cercariae. The minimum prepatent period of 17–19 days and 17–20 days observed among snails maintained at 30–31 °C and 32 °C, respectively. Longest prepatent period of 106–113 days observed among snails maintained at 18 °C. A hyperbolic function developed to determine the relationship between temperature and the length of the prepatent period estimated 15.3 °C as the temperature threshold at which parasite development within the *Bu. truncatus* theoretically comes to a standstill.
Pflüger [43]	To determine the effect of temperature on the length of the prepatent period in infected *Bi. glabrata* snails	*Biomphalaria glabrata*	Laboratory experiment	Nearly all snails maintained at 16 °C died during the prepatent period. At this temperature, the prepatent period approximated to take more than 130 days. Minimum prepatent period of 15 days observed among snails maintained at 32 and 33 °C. A hyperbolic function developed to determine the relationship between temperature and the length of the prepatent period estimated 14.2 °C as the temperature threshold at which parasite development within the *Bi. glabrata* theoretically comes to a standstill.
Mofolusho and Benson [44]	To access the influence of acclimatization (to laboratory conditions) on the fecundity and fertility of field collected *Bi. pfeifferi*	*Biomphalaria pfeifferi* (Krauss, 1848)	Laboratory experiment	The mean number of eggs per egg mass in laboratory bred snails was higher than in acclimatized field collected snails. The survival rate of acclimatized snails is lower than that of laboratory bred snails.
Joubert et al. [45]	To determine the survival of *Bu. africanus* (Krauss), *Bu. globosus* (Morelet) and *Bi. pfeifferi* (Krauss) at constant low temperatures (0 °C to 8 °C)	*Bulinus africanus* (Krauss), *Bulinus globosus* (Morelet) and *Biomphalaria pfeifferi* (Krauss)	Laboratory experiment	Snail survival reduced with a reduction in temperature. Lower temperatures more unfavourable for *Bi. pfeifferi* than *Bu. africanus* or *Bu. globosus*.
Barbosa et al. [46]	To determine the effect of seasonal temperature variation on egg production during the year	*Biomphalaria glabrata*	Laboratory experiment	The snail reproductive rate was observed to vary inversely with temperature. Snail reproductive rate was highest when temperature was around 19.9 °C. The reproductive rate as affected reduced as temperature rose to 24 °C during summer. The number of eggs per mass was observed to be maximal when temperature was around 22 °C observed during autumn.
O’keeffe [47]	To evaluate the effect of seasonal climatic changes on the natural populations of *Bu. globosus*	*Bulinus globosus*	Field experiment	The snail intrinsic growth rate reduces with rise in temperature beyond 28.5 °C. The per capita recruitment rate of snails increased with temperature. It was maximal at 20.6 °C, beyond which it started reducing.
Woolhouse and Chandiwana [48]	To determine the factors that influence the abundance of *Bu. globosus* in space and time for a critical assessment of the possible effectiveness of different snail control strategies	*Bulinus globosus*	Field experiment	Snails maintained in cages at 14–16 °C, 21–22 °C and 22.5–23.5 °C. Growth rate increased with rise in temperature.
Mangal et al. [49]	To determine the impact of temperature on the worm burden and prevalence of schistosomiasis for optimal disease control strategies	Non specific *Biomphalaria* spp.	Modelling	The mean worm burdens maximal at 30 °C and reduces sharply at 35 °C. Schistosomiasis prevalence was stable in the temperature range of 20–35 °C. Parasite survival optimum at 20 °C thus more effective to carry out disease control measures during times of increased parasite survival.
McCreesh and Booth [50]	To simulate all temperature-sensitive stages of *S. mansoni* and the life cycle of its intermediate host snail *Bi. pfeifferi*	*Biomphalaria pfeifferi*	Modelling	Model developed for transmission sites such as rivers and lakes. In the lake and river scenarios, infection among humans was modelled to be high at 15–19 °C and 20.5–25 °C, respectively. Outside the temperature range of 14.0–31.5 °C, snail survival reduces greatly. The prepatent period reduces with increasing time. For example, it reduces from 130 days at 14.0 °C to 18 days at 32 °C. In the lake and river scenarios, temperature ranges above 15–18 °C and 15–20 °C, respectively led to a reduction in the number of infected snails.
Ngarakana-Gwasira et al. [51]	To develop an epidemiological model for improved predictions of the impact of climatic factors on the dynamics and variation of schistosomiasis intensity in Zimbabwe	Non-specific snail species	Modelling	The temperature ranges of 18–28 °C was observed to be ideal for schistosomiasis transmission. The optimal schistostosoma transmission temperature was around 23 °C. This was supported by the reproductive number which increased linearly with temperature and reached it maximum at 23 °C. Thereafter, it started reducing. Infection among snails higher at 22 °C compared to temperature of 20 °C and 25 °C while it dies out at 30 °C.

**Table 2:** Some important terms that have been used in the review.

Term	Meaning
Growth	The increase in the shell height of the IH snails [41]
Fecundity	A measure of the fertility of the organisms expressed as number of egg masses laid [36,40,41]
Reproductive rate	The mean number of offspring that the snail can produce during its lifetime [38,40]
